# Diagnostic Performance of ADNEX Model and IOTA Simple Rules in Differentiating Malignant from Benign Adnexal Masses When Assessed by Non-Expert Examiners

**DOI:** 10.3390/jcm14082776

**Published:** 2025-04-17

**Authors:** Ammaraporn Chankrachang, Worashorn Lattiwongsakorn, Charuwan Tantipalakorn, Theera Tongsong

**Affiliations:** Department of Obstetrics and Gynecology, Faculty of Medicine, Chiang Mai University, Chiang Mai 50200, Thailand

**Keywords:** adnexal mass, ADNEX model, IOTA Simple Rules, ovarian tumor, ultrasound

## Abstract

**Objective**: The aim of the study was evaluate the diagnostic performance of the ADNEX model performed by non-expert examiners in differentiating between benign and malignant adnexal masses and to compare its performance with the IOTA Simple Rules. **Methods**: This diagnostic study was a secondary analysis based on a prospectively collected database of gynecological patients scheduled for elective surgery for adnexal masses. Preoperative ultrasound was performed within 24 h before surgery by second- and third-year gynecology residents who had completed a 20 h training course on adnexal mass ultrasound. Ultrasound data, CA-125 levels, and demographic information were reviewed and validated. Malignancy risk assessment using the IOTA Simple Rules was based on M-rules and B-rules, while risk calculations with the ADNEX model were conducted using an online application. **Results**: The area under the curve (AUC) for the ADNEX model was 0.958 (95% CI: 0.936–0.980), which was significantly higher than that of the IOTA Simple Rules at 0.886 (95% CI: 0.840–0.931; *p* < 0.001). Using a 10% cutoff, the ADNEX model demonstrated a sensitivity of 93.9% and a specificity of 81.0%, while the IOTA Simple Rules had a sensitivity of 84.0% and a specificity of 93.1%. The AUCs of the ADNEX model did not significantly differ when center status was considered (oncologic vs. non-oncologic centers). **Conclusions**: The ADNEX model, using a 10% cutoff, exhibited superior efficacy in differentiating benign from malignant adnexal masses compared with the IOTA Simple Rules. Furthermore, the sensitivity and specificity of the ADNEX model did not significantly differ between oncologic and non-oncologic centers. Both methods demonstrated high diagnostic accuracy, even when performed by non-expert examiners, suggesting that their diagnostic performance is reproducible and applicable in general clinical practice by general practitioners and gynecologists.

## 1. Introduction

Accurately predicting whether an adnexal mass is benign or malignant is crucial for surgical planning. This includes determining the need for complete surgical staging, consulting specialists in ovarian cancer surgery, and counseling patients at higher risk of complications, such as older individuals or those with pre-existing health conditions. Early risk assessment may also help avoid unnecessary surgery in patients with benign ovarian cysts or facilitate the use of minimally invasive procedures in cases of benign tumors. Currently, various methods are being explored for the early diagnosis and screening of ovarian cancer, including pelvic examinations, tumor markers, and ultrasound imaging. Transvaginal ultrasound, in particular, has been widely utilized to improve diagnostic accuracy, as it enables the visualization of even small pathologies and enhances the differentiation between benign and malignant tumors. Moreover, this method is safe, relatively simple, well accepted by patients, and time-efficient. However, the diagnostic performance of these techniques varies across studies and testing systems, as multiple classification systems are in use.

Ultrasound-based assessment of ovarian cancer risk employs several widely used methods, especially the IOTA Simple Rules developed by the International Ovarian Tumor Analysis (IOTA) group and the ADNEX model. The IOTA Simple Rules utilize ultrasound to evaluate the characteristics of ovarian masses and determine the likelihood of malignancy, based on five ultrasound features indicating malignancy (M-features) and five features suggesting a benign lesion (B-features) [1,2,3,4]. A key advantage of this method is that it does not require tumor marker values for risk assessment. However, its primary limitation is that approximately 15–24% of cases yield inconclusive results [2,5,6,7]. The ADNEX model is an extension of the IOTA framework that incorporates clinical data to assess the risk of benign tumors, borderline tumors, and malignant ovarian cancer [8]. It is available on the IOTA website (http://www.iotagroup.org/adnexmodel, assessed on 27 February 2024) and allows clinicians to input patient data into a user-friendly online platform. The model evaluates nine clinical parameters: patient age (years), serum CA-125 level (U/mL), type of treatment center (oncology or general), maximum tumor diameter, maximum width of the solid tumor component, presence of more than 10 cystic locules, number of papillary projections (0, 1, 2, 3, or more than 3), presence of acoustic shadowing, and presence of ascites. The system provides percentage-based risk estimates for benign tumors, borderline tumors, early-stage cancer, advanced-stage cancer, and metastatic cancer. The ADNEX model has demonstrated superior discriminatory performance and clinical utility in differentiating malignant from benign adnexal masses compared to CA125, HE4, the Risk of Malignancy Algorithm (ROMA) score, and the Risk of Malignancy Index (RMI) [9,10,11]. At cut-off 10% probability, it provides a sensitivity of 92–98% and specificity of 75–88% [9,12,13,14]. Additionally, it provides fair to excellent discrimination among four subtypes of ovarian malignancy based on subgroup staging [14]. Additionally, the ADNEX model can be applied to resolve inconclusive results from the Simple Rules when used in a two-step strategy, with the IOTA Simple Rules or IOTA benign simple descriptor or benign simple descriptors serving as the first-line approach [13,15]. To date, several systems have been proposed to differentiate between benign and malignant adnexal masses. Currently, two systems have gained increasing popularity [16,17]: (1) the Ovarian-Adnexal Reporting and Data System (O-RADS) ultrasound risk stratification and management system, developed by the American College of Radiology (O-RADS US working group), which is commonly used in the United States, and (2) the IOTA systems, including the IOTA Simple Rules and the ADNEX model, which are more widely adopted, particularly in Europe. The IOTA systems have been more extensively studied and published. Owing to its accuracy and ease of use, the ADNEX model has gained widespread acceptance in clinical practice.

As most of these diagnostic systems have been primarily studied within research center networks, there remains a paucity of studies in other populations outside western countries. Furthermore, external validation assessing their effectiveness in real-world clinical practice is limited, and their reproducibility in general practice or when used by non-expert examiners has rarely been evaluated. To address this gap, the research team primarily aimed to compare the diagnostic performance of the IOTA Simple Rules and ADNEX model in distinguishing between benign and malignant adnexal masses when used by non-expert examiners. Unlike most previous studies, we focused on the implementation of the ADNEX model in real-world clinical practice, where it is applied by general gynecologists rather than by experts in a controlled research setting. We aimed to determine whether the high diagnostic performance reported in most studies can be reproduced by non-expert practitioners. We hypothesized that, due to its relative simplicity, the model may indeed be reproducible. If so, the ADNEX model could have a significant impact on improving the accuracy of differentiating between benign and malignant adnexal masses. This would be particularly beneficial in routine practice, enabling general gynecologists to better identify cases requiring referral to tertiary care centers while confidently managing benign cases within their own local hospitals.

## 2. Materials and Methods

Study design: A secondary analysis of a diagnostic study was conducted on gynecologic patients undergoing elective surgery for ovarian tumors or adnexal masses at Maharaj Nakorn Chiang Mai Hospital, a tertiary medical teaching center in Chiang Mai, Thailand. The study was ethically approved by the Institutional Review Board (Research Committee 4, Faculty of Medicine, Chiang Mai University, Thailand) under Research ID: OBG-2567-0076. This analysis was based on a prospective database on adnexal mass assessment by non-expert examiners that was established as part of our project. Data were prospectively collected and included comprehensive ultrasound and clinical variables of adnexal masses, with examinations performed by residents under supervision. Each case met the predefined inclusion criteria for subsequent analysis. Additionally, demographic data and clinical information, including age, parity, menopausal status, and CA-125 levels, were prospectively recorded. Portions of the dataset were utilized in previous studies [7,18].

Study population: Patients scheduled for surgery for adnexal masses were recruited and underwent transabdominal and transvaginal ultrasound within 24 h before the operation. During database development, patients were counseled and invited to participate with the study. All of the patients provided written informed consent.

Ultrasound Diagnostic Model and Strategies: Ultrasound examinations were performed by second- or third-year obstetrics and gynecology residents, who were considered non-expert examiners. These residents had completed a 20 h training course on the IOTA Simple Rules and ADNEX model, which included lectures and ultrasound clip-based learning. The ultrasound examinations were conducted under the supervision of experienced staff members using GE Voluson E8 and Voluson E10 scanners (GE Medical Systems, Zipf, Austria), equipped with an abdominal transducer operating at 2–5 MHz and a transvaginal transducer operating at 5–7.5 MHz, with a scan angle of 120 degrees. The ultrasound results were interpreted and recorded by the residents in the database record form before surgery. The recorded ultrasound parameters included characteristics indicative of malignant tumors (M-rules) and benign tumors (B-rules) based on the IOTA Simple Rules, as described previously [2]. Also, parameters assessed for the ADNEX model included [14], for example, tumor component (solid/cystic), surface characteristics (irregular/smooth), the presence or absence of ascites, papillary structures, tumor morphology (unilocular/multilocular), number of locules, largest tumor diameter, color (blood flow) score, largest solid component diameter, and the presence of acoustic shadows.

Reference Standard: The final diagnosis, considered the reference standard, was based on pathological examination of surgical specimens in most cases. In certain cases involving benign conditions without available pathological specimens, such as tubo-ovarian abscesses, pseudocysts, or hemorrhagic cysts, in some cases, the diagnosis was made intraoperatively by the surgical team.

Primary endpoint: The primary outcome was the classification of adnexal masses into malignant or benign groups based on the reference standard. Borderline tumors or those with low malignant potentials were categorized as malignant.

Database assessment: All records were consecutively reviewed and validated based on the inclusion criteria. Full digital medical records were also reviewed to confirm the baseline characteristics, with clinical data including tumor marker examination data system and pathological reports. The inclusion criteria were as follows: (1) Patients diagnosed with an adnexal mass or ovarian tumor detected by prior ultrasound or pelvic examination. (2) Patients with no known diagnosis of the adnexal mass before surgery, including those without prior laparoscopic examination or previous surgical intervention. (3) Patients with available CA-125 test results obtained within three months before surgery. The exclusion criteria were as follows: (1) Patients who underwent surgery more than 24 h after the ultrasound examination. (2) Patients with incomplete ultrasound evaluation. (3) Patients without available CA-125 results. A digital case record form was used to document clinical and ultrasound parameters. Data were securely collected, and hospital numbers and patient names were replaced with unique identifiers to ensure patient privacy and confidentiality. Upon data retrieval and assessment, ultrasound variables and clinical data were validated to determine the malignancy risk of adnexal masses using the IOTA Simple Rules [2] and risk calculations from the ADNEX model [14]. The risk assessment was conducted using the online ADNEX model application by the first author (AC). The ADNEX model is available online at www.iotagroup.org/adnexmodel/, assessed on 27 February 2024.

Statistical analysis: Statistical analyses were performed using the Statistical Package for the Social Sciences (SPSS) software, version 26.0 (IBM Corp., Released 2019; IBM SPSS Statistics for Windows, Version 26.0, IBM Corp., Armonk, NY, USA). In the analysis, borderline ovarian tumors were grouped with ovarian cancer as the malignant group. Descriptive statistics for demographic data were presented as number (percentage), mean ± standard deviation, or median (interquartile range), as appropriate. Comparative analyses were conducted using the chi-square test for categorical variables and either Student’s *t*-test or the Mann–Whitney U test for continuous variables. The diagnostic performance of the IOTA Simple Rules and the ADNEX model was assessed using receiver operating characteristic (ROC) curve analysis, with sensitivity (at the cutoff value of 10% for ADNEX model), specificity, and predictive values calculated. Comparisons of area under the curve (AUC) values were performed using the conditional risk method for pairwise analysis. A *p*-value of <0.05 was considered statistically significant.

## 3. Results

**Figure 1:**
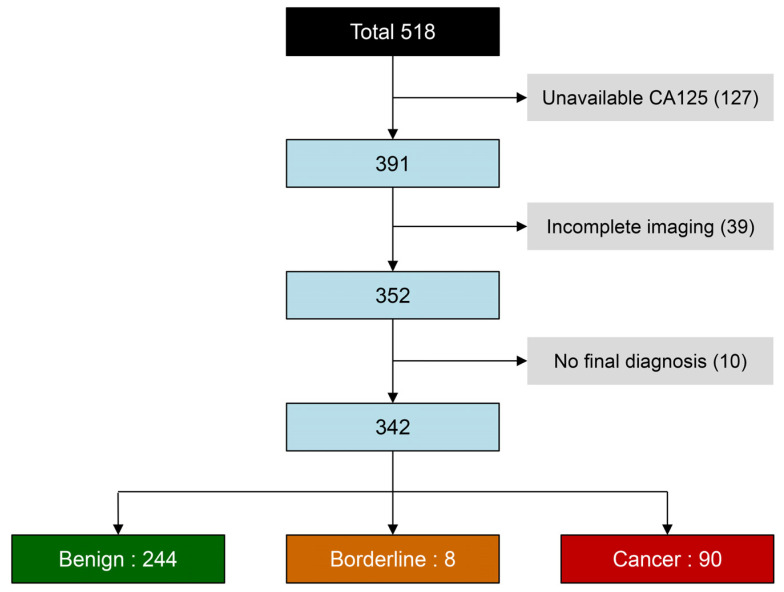
Flowchart of the patient recruitment and exclusion.

A total of 518 adnexal masses from patients undergoing elective surgery for adnexal masses were included in the study, as shown in Figure 1. Of these, 176 cases were excluded due to unavailable CA-125 levels, incomplete imaging, or the absence of a definitive diagnosis. The remaining 342 cases were included in the analysis, comprising 244 benign masses (71.3%) and 98 malignant masses (28.7%), including 8 borderline tumors.

The baseline characteristics of the two groups are presented in Table 1. The mean age of patients in the malignant group was significantly higher than that of those in the benign group, and parity was also significantly greater in the malignant group. Additionally, the proportion of postmenopausal women was higher in the malignant group compared to the benign group.

Regarding tumor characteristics, the CA-125 levels, median tumor diameter, percentage of cases with a solid component, number of locules, number of papillations, and presence of ascites were significantly higher in the malignant group. In contrast, the number of cases with acoustic shadowing was significantly higher in the benign group (*p* < 0.001), as shown in Table 2.

Regarding the final diagnosis of adnexal masses, endometrioma was the most common, with 82 cases (24%), followed by mature cystic teratoma in 49 cases (14.3%) and serous cystadenoma in 27 cases (7.9%), as shown in Table 3. In the malignant group, the most common diagnosis was endometrioid carcinoma, with 24 cases (7%), followed by mucinous cystadenocarcinoma in 21 cases (6.1%), serous cystadenocarcinoma in 20 cases (5.8%), and other diagnoses, as shown in Table 3.

The diagnostic performance of the ADNEX model, using a cut-off of >10%, yielded a sensitivity of 93.9% (95% CI: 87.1–97.7), specificity of 81% (95% CI: 75.5–85.7), and an AUC of 0.958 (95% CI: 0.932–0.984), as presented in Table 4. The diagnostic performance of IOTA Simple Rules was a sensitivity of 84% (95% CI: 73.7–91.4), specificity of 93.1% (95% CI: 88.8–96.2), and an AUC of 0.886 (95% CI: 0.840–0.931). When comparing the diagnostic performance of the ADNEX model (using a cut-off of >10%) with that of IOTA Simple Rules, the ADNEX model demonstrated higher sensitivity and AUC but lower specificity and positive predictive value (PPV), as shown in Table 4.

**Figure 2:**
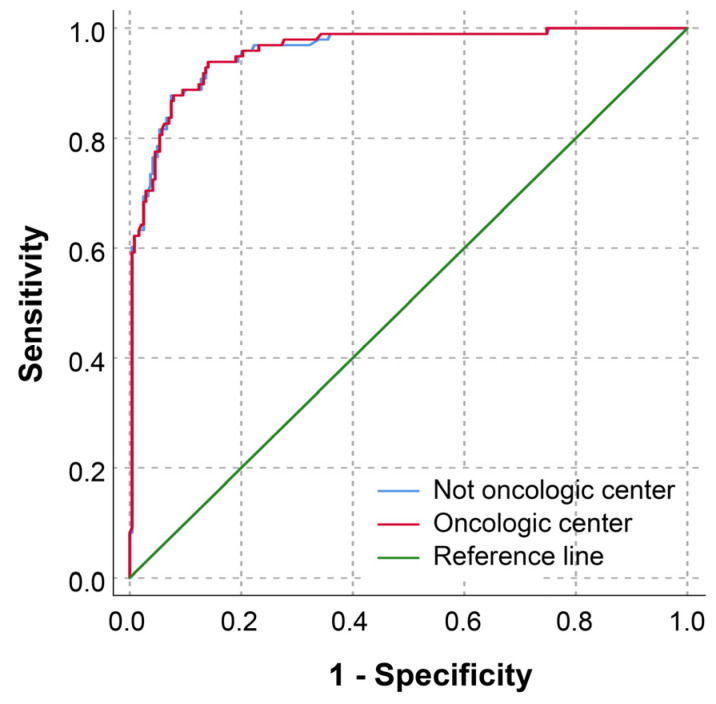
Receiver operating characteristics (ROC) curves of the ADNEX model in differentiating benign and malignant adnexal masses, comparing between that performed at non-oncologic center and oncologic center. AUC (95% CI) of non-oncologic vs. oncologic center: 0.957 (0.935–0.980) vs. 0.958 (0.936–0.980); *p*-value: 0.585.

The receiver operating characteristic (ROC) curves of the ADNEX model for differentiating benign and malignant adnexal masses, comparing those performed at a non-oncologic center and an oncologic center, are similar, as shown in Figure 2. The AUC (95% CI) for the non-oncologic center is 0.957 (0.935–0.980), whereas the AUC for the oncologic center is 0.958 (0.936–0.980), with a *p*-value of 0.585.

## 4. Discussion

The key insights from this study are as follows: (1) The IOTA Simple Rules and the ADNEX model are highly effective in distinguishing between benign and malignant adnexal masses, with the ADNEX model demonstrating a slightly but significantly superior performance, as evidenced by an area under the curve (AUC) of 0.958. (2) The diagnostic performance of these techniques is reproducible, even when assessed by non-expert examiners in an external validation setting. (3) Using the conventional cut-off, the ADNEX model exhibits significantly higher sensitivity but lower specificity than the IOTA Simple Rules. (4) About 16% of cases yield inconclusive results with the IOTA Simple Rules, whereas the ADNEX model can be applied in all cases.

Unlike most previous studies, ultrasound examinations in this study were performed by non-expert gynecologists, i.e., in-training obstetric/gynecologic residents, as part of an external validation process. Although our center is a tertiary healthcare facility, the ultrasound examiners were non-experts, which may better reflect the setting of a non-oncologic center. Consequently, our findings suggest that the performance of the ADNEX model is likely reproducible by general practitioners with limited experience, regardless of whether they practice in an oncologic or non-oncologic center. Based on the AUCs, the diagnostic performance of the ADNEX model does not differ significantly between these settings. Therefore, this study indicates that, in addition to non-expert examiners, the ADNEX model is likely to be reliably applied in general practice when used by general practitioners in non-specialized hospitals. The non-expert examiners in this study may not perfectly represent general gynecologists. However, it is likely that general gynecologists could apply the ADNEX model with comparable or even superior accuracy, given their higher qualifications and greater experience in gynecologic practice. Additionally, it is noteworthy that the ultrasound machines used in this study were not significantly different from those commonly used in routine practice in our country. They were of average quality rather than high-end or advanced models. Therefore, the results demonstrated in this study are expected to be generalizable and reproducible in typical clinical settings. In comparison, the sensitivity among non-expert examiners in our study was comparable to or slightly lower than that reported in most previous studies, which demonstrated sensitivities ranging from 92% to 98% [9,12,13,14]. Although the specificity in our study remained relatively high, it was slightly lower than that reported in most previous studies, which ranged from 75% to 88% [9,12,13,14]. In other words, the false-positive rate was somewhat higher. This may be attributed to a tendency among examiners to classify uncertain benign cases as malignant, favoring a worst-case scenario. For example, amorphous masses in atypical dermoid cysts may be misinterpreted as solid components suggestive of malignancy. Nevertheless, we believe that this issue can be mitigated through increased experience and continued practice in real clinical settings.

Typically, the prevalence of malignant cases in oncologic centers is significantly higher than in general hospitals. Statistically, a higher prevalence increases the positive predictive value but does not affect sensitivity or specificity. Therefore, the characteristics of the center do not influence the validity of a diagnostic test or its area under the curve (AUC). However, physicians in oncologic centers are more frequently exposed to ultrasound imaging of malignant cases and tend to have greater experience, which may influence diagnostic performance. In this study, although conducted in an oncologic center, the ultrasound examinations were performed by inexperienced examiners. As a result, the findings likely reflect those of a non-oncologic center, which may explain the slightly lower performance observed in our study compared to some previous studies. For example, Poonyakanok et al. [9] demonstrated that the ADNEX model, using a cut-off of >10%, yielded a sensitivity of 98.4% and a specificity of 87.2%.

The ADNEX model demonstrates slightly higher effectiveness than the IOTA Simple Rules, likely due to the added value of clinical characteristics incorporated into the model. Additionally, inconclusive results were observed in approximately 16% of cases with the IOTA Simple Rules, consistent with other previous studies [1,2,7,18]. Notably, the IOTA Simple Rules missed more than 15% of malignant masses, whereas the ADNEX model yielded false-positive results in nearly 20% of cases, potentially leading to overtreatment. However, from a clinical perspective, a high sensitivity with an acceptable false-positive rate is often preferred. Therefore, in clinical practice, the ADNEX model should be prioritized over the IOTA Simple Rules. Nevertheless, for sonographers or radiologists who do not have access to clinical information, the IOTA Simple Rules remain a suitable option due to their effectiveness and exclusive reliance on imaging characteristics.

Notably, blood flow assessment is not included in the ADNEX model despite its usefulness in distinguishing between malignant and benign adnexal masses. A previous study demonstrated that increased blood flow, assessed using a simple method, is a component of the IOTA model with a high likelihood ratio [8]. Therefore, future research should explore whether incorporating blood flow assessment into the ADNEX model enhances its diagnostic performance. This study highlights that the ADNEX model is associated with a relatively high false-positive rate (nearly 20%) and may benefit from modification. Incorporating blood flow assessment could potentially improve its performance. Notably, this addition is straightforward, can be performed during the same examination, and does not require additional cost or effort, as color flow mapping is available on nearly all modern ultrasound machines. Furthermore, the ADNEX model currently incorporates only age as a clinical risk factor, while other important factors, such as a family history of ovarian cancer, history of hormonal contraceptive use, and menopausal status, are not considered. Future studies integrating these clinical variables may further enhance the model’s predictive accuracy without additional cost or complexity.

The strengths of this study include the following: (1) an adequate sample size; (2) external validation in a population distinct from Western women, which have been the focus of most previous studies [1,14,19]; and (3) assessment by non-expert examiners, signifying that both the ADNEX model and the IOTA Simple Rules are likely effective when applied by general gynecologists or general practitioners. The limitations of this study are as follows: (1) The diagnostic performance of the ADNEX model without CA-125 was not evaluated, limiting its applicability in settings where CA-125 testing is unavailable. (2) The model was not assessed for its ability to differentiate cancer types or stages, as was done in the original study [14]. (3) This study included only cases that underwent surgery and had a definitive final diagnosis. Consequently, the findings may be applicable primarily to preoperative patients. (4) Inter-rater variability among the examiners and the assessment of the learning curve were not evaluated; therefore, the reproducibility and generalizability of the findings should be interpreted with caution. However, this limitation is unlikely to compromise the results or conclusions, as the examiners’ performance is expected to improve in actual clinical practice with increased experience and progression along their learning curves.

## 5. Conclusions

Both the ADNEX model and the IOTA Simple Rules are highly effective in distinguishing benign from malignant adnexal masses, even when performed by non-expert examiners. This suggests that their diagnostic performance is likely reproducible and can be clinically applied by general practitioners or general gynecologists. Between the two techniques, the ADNEX model is more favorable due to its higher sensitivity and acceptable specificity and the relatively high rate of inconclusive results associated with the IOTA Simple Rules.

## Figures and Tables

**Table 1:** Baseline characteristics of the patients of the two groups.

	Benign (n: 244)	Malignant (n: 98)	*p*-Value
Age (years); mean ± SD	39.8 ± 11.0	45.4 ± 14.7	<0.001
Parity			0.008
0	109 (44.7%)	52 (53.1%)	
1	87 (35.7%)	17 (17.3%)	
2	36 (14.9%)	21 (21.4%)	
3 or more	12 (4.9%)	8 (8.2%)	
Menopausal status			<0.001
Premenopausal	205 (84.0%)	60 (61.2%)	
Postmenopausal	39 (16.0%)	38 (38.8%)	

**Table 2:** Tumor characteristics of the two groups.

	Benign (n: 244)	Malignant (n: 98)	*p*-Value
CA 125 levels; median (IQR)	40.9 (25.9; 61.8)	403.3 (122.3; 707.6)	<0.001
Maximal diameter (cm); median (IQR)	9.2 (7.3; 13.1)	11.9 (9.1; 16.6)	<0.001
Solid part; n (%)	42 (17.2%)	84 (85.7%)	<0.001
Solid part diameter (cm); median (IQR)	0.0 (0.0; 1.0)	6.6 (3.1; 9.9)	<0.001
Number of locules; median (IQR)	1 (1; 4)	5 (3; 10)	<0.001
Number of papillations; n (%)			<0.001
0	218 (89.3%)	48 (49.0%)	
1	11 (4.5%)	1 (1.0%)	
2	5 (2.0%)	2 (2.0%)	
3	2 (0.8%)	4 (4.1%)	
4 or more	8 (3.3%)	43 (43.9%)	
Acoustic shadow; n (%)	74 (30.3%)	11 (11.2%)	<0.001
Ascites; n (%)	3 (1.2%)	14 (14.3%)	<0.001

**Table 3:** Final diagnosis of the adnexal mass.

Final Diagnosis	Number	Percentage
Benign group	244	
Mature cystic teratoma	49	14.3
Benign Brenner tumor	3	0.9
Endometrioma	82	24.0
Fibroma	10	2.9
Hemorrhagic cyst	3	0.9
Mucinous cystadenoma	19	5.6
Pedunculated leiomyoma	16	4.7
Pseudocyst	20	5.8
Serous cystadenoma	27	7.9
Simple epithelial cyst	9	2.6
Tubo-ovarian abscess	6	1.8
Malignant group	98	
Serous tumor of low malignant potentials	3	1.8
Mucinous tumor of low malignant potentials	5	1.8
Clear cell carcinoma	7	2.0
Dysgerminoma	2	0.6
Endometrioid carcinoma	24	7.0
Immature teratoma	5	1.5
Mucinous cystadenocarcinoma	21	6.1
Serous cystadenocarcinoma	20	5.8
Sex-cord stromal tumor	4	1.2
Yolk sac tumor	2	0.6
Metastatic carcinoma	3	0.9
Other cancers	2	1.2
**Total**	**342**	**100**

**Table 4:** Diagnostic performance of the IOTA Simple Rules and the ADNEX model in predicting adnexal malignant tumor.

	Final Diagnosis		Diagnostic Performance				
	Benign	Malignant	Sens (95% CI)	Spec (95% CI)	PPV (95% CI)	NPV (95% CI)	AUC (95% CI)
**Simple rules**							
Negative	190	12	84.0% (73.7–91.4%)	93.1% (88.8–96.2%)	81.8% (71.4–89.7%)	94.1% (89.9–96.9%)	0.886 * (0.840–0.931)
Positive	14	63					
**Total (n)**	204	75					
**ADNEX model cut-off 10%**							
Negative	196	6	93.9% (87.1–97.7%)	81.0% (75.5–85.7%)	66.7% (58.1–74.5%)	97.0% (93.6–98.9%)	0.958 * (0.932–0.984)
Positive	46	92					
**Total (n)**	**242**	**98**					

* *p*-value < 0.001 (Sens: sensitivity; Spec: specificity; PPV: positive predictive value; NPV: negative predictive value; AUC: area under curve).

## Data Availability

The datasets analyzed in the current study are available from the corresponding author upon reasonable request.
